# Phylogenomics of Dengue Virus Isolates Causing Dengue Outbreak, São Tomé and Príncipe, 2022

**DOI:** 10.3201/eid3002.231316

**Published:** 2024-02

**Authors:** Lazismino Lázaro, Doris Winter, Katia Toancha, Adjaia Borges, Anabela Gonçalves, Asmiralda Santos, Marcos do Nascimento, Nilton Teixeira, Yardlene Sacramento Sequeira, Anery Katia Lima, Bakissy da Costa Pina, Andreza Batista de Sousa, Jürgen May, Rosa Maria Afonso Neto, Kathrin Schuldt

**Affiliations:** National Reference Laboratory for Tuberculosis and Emerging Diseases, Ministry of Health, São Tomé, São Tomé and Príncipe (L. Lázaro, K. Toancha, A. Borges, A. Gonçalves, A. Santos, M. do Nascimento, N. Teixeira, Y. Sacramento Sequeira, A.K. Lima, R.M. Afonso Neto);; Bernhard Nocht Institute for Tropical Medicine, Hamburg, Germany (D. Winter, J. May, K. Schuldt);; National Emergency Operating Center, Ministry of Health, São Tomé (B. da Costa Pina);; National Surveillance Department, Ministry of Health, São Tomé (A. Batista de Sousa);; German Center for Infection Research, Hamburg–Lübeck–Borstel–Riems, Germany (J. May);; University Medical Center Hamburg–Eppendorf, Hamburg (J. May)

**Keywords:** dengue virus, outbreak, genomic surveillance, viruses, arboviruses, phylogenomics, Nanopore long read sequencing, São Tomé and Príncipe

## Abstract

We determined that the dengue outbreak in São Tomé and Príncipe during 2022 was caused by dengue virus serotype 3 genotype III. Phylogenomic analyses showed that the outbreak strain was closely related to the newly identified GIII-American-II lineage and that the virus probably was introduced from the Americas.

Globally, dengue case numbers have increased dramatically over recent decades; an estimated 96 million clinical dengue cases per year have been reported in >100 countries (*1*). Dengue is an acute febrile disease that can evolve into a severe life-threatening disease. Dengue is caused by an infection with the dengue virus (DENV), a member of the family *Flaviviridae*, and has 4 different serotypes (DENV-1–4) and distinct infection dynamics (*2*).

In 2022, São Tomé and Príncipe, an island state with ≈210,000 inhabitants in the Gulf of Guinea in sub-Saharan Africa, reported the occurrence of dengue cases in the country. During epidemiologic weeks 15–50 in 2022, a total of 1,152 dengue fever cases confirmed by positive rapid diagnostic tests (RDTs) were reported. The first cases were reported April 15, and case numbers peaked at 178 notifications in week 24 (Appendix Figure). Among the 1,152 RDT-confirmed cases, the most frequent observed symptoms were fever (92%), headache (78%), and myalgia (38%). A total of 144 (12.5%) persons were admitted to the hospital (Appendix Table 1), and 8 persons died from infection with the virus. The presumptive index patient was described as a 27-year-old man from São Tomé and Príncipe who had traveled to the island of Guadeloupe before arriving in São Tomé on March 26, 2022, and whose onset of symptoms occurred on April 4, 2022 (*3*). A previous study analyzed the seroprevalence of DENV antibodies in the São Tomé and Príncipe population. In that study, 31 of 78 tested pregnant women were found to be seropositive for DENV, indicating that the country’s population might have experienced exposure to the virus before 2003–2004, during which the collection of the analyzed serum samples took place (*4*).

This study was approved by the Health Ethics Committee for Scientific Research at the Ministry of Health of STP (approval no. 015B/2022). During May 6–16, 2022, we collected 7 plasma samples from dengue RDT–positive patients in São Tomé and Príncipe (Appendix Table 2). All 7 infections were confirmed by real-time PCR, and subtyping revealed the presence of DENV-3 (Appendix Table 2). Long-read whole-genome sequencing and subsequent assembly (reference strain GenBank accession no. NC_001475) resulted in 48–64,440 assembled reads (Appendix Table 3) with an average depth of coverage of 4–4,148× (Appendix Figure 2). We classified all 7 isolates as DENV-3 genotype III (GIII) by using a flavivirus genotyping tool (*5*) with bootstrap support of 100.

To study the evolutionary relationship of the virus isolates from São Tomé and Príncipe, we included 4 reconstructed genomes with best assembly results (>10 kb, genome coverage >98%, depth of coverage >250×) in a phylogenomic analysis together with 1,168 DENV-3 GIII genomes (Appendix Table 4) sampled worldwide. All 1,172 sequences passed the IQ-TREE2 composition test. The best-fitting evolutionary model according to Bayesian information criterion (BIC) was the general time-reversible plus empirical frequencies plus invariate sites plus FreeRate model. The reconstructed consensus tree revealed that the newly sequenced DENV-3 isolates from São Tomé and Príncipe clustered with and are closely related to the new monophyletic clade consisting of 218 DENV-3 sequences detected in the Americas during 2022–2023 (Figure). A recent study by Naveca et al. (*6*) demonstrates that this new lineage (GIII-American-II lineage) was most likely introduced to Cuba from the Indian subcontinent in 2019 (*6*). Consistent with their findings, our consensus tree (Figure) shows 3 DENV-3 sequences collected in India in 2018 as part of the next bigger clade comprising the GIII-American-II lineage and the 4 isolates from São Tomé and Príncipe. 

**Figure:**
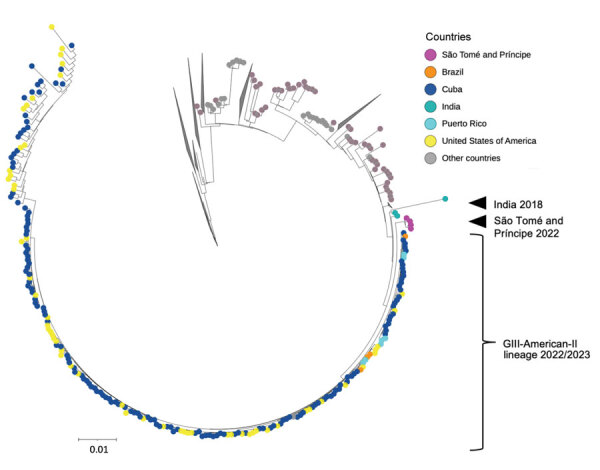
Reconstructed consensus tree of newly sequenced dengue virus serotype 3 isolates from São Tomé and Príncipe. The isolates clustered with and are closely related to a new monophyletic clade consisting of 218 dengue virus serotype 3 sequences detected in the Americas during 2022–2023. To improve visualization, several clades have been collapsed. Scale bar indicates nucleotide substitutions per site.

Because the index patient reportedly had traveled to Guadeloupe before arriving in São Tomé and Príncipe, the likely scenario of virus importation is that after the introduction of the DENV-3-GIII lineage from Asia to America during 2018–2020, the virus might have circulated in the region, and from there it was introduced to São Tomé and Príncipe in 2022. Although we did not conduct formal phylogeographic analysis as part of this study, 2 points support our conclusions: the epidemiologic information that the index patient visited Guadalupe; and the results of the previous study from Brazil, describing the new DENV-3, GIII-American-II lineage and how it arose in America (*6*). Thus, our results suggest that the São Tomé and Príncipe outbreak originated from the new American lineage.

According to surveillance data of the Pan American Health Organization, Guadeloupe has experienced yearly dengue outbreaks since 2018, and in the year 2020, the serotypes 1–3 were detected (*7*). Unfortunately, no information is available on the DENV serotype or genomic sequences on the DENV circulating in 2022 in Guadeloupe, and only sparse information is available on dengue cases from countries in Africa.

The results of our study corroborate a possible global expansion of the new DENV-3 GIII-American-II clade previously described by Naveca et al. (*6*). Furthermore, finding this American lineage in Africa reinforces the importance of genomic surveillance of DENV in countries at risk for future outbreaks.

Appendix Additional information about phylogenomics of dengue virus isolates causing dengue outbreak, São Tomé and Príncipe, 2022.
