# Supplementary material for: Phylogenomics of Dengue Virus Isolates Causing Dengue Outbreak, São Tomé and Príncipe, 2022
Source: Emerg Infect Dis. 2024 Feb;30(2):384–6. doi: 10.3201/eid3002.231316 (PMC10826765; doi:10.3201/eid3002.231316)
Supplement: Appendix — Additional information about phylogenomics of dengue virus isolates causing dengue outbreak, São Tomé and Príncipe, 2022. [file 23-1316-Techapp-s1.pdf]

Article DOI: https://doi.org/10.3201/eid3002.231316

EID cannot ensure accessibility for supplementary materials supplied by authors. Readers who have difficulty accessing supplementary content should contact the authors for assistance.

Phylogenomics of Dengue Virus Isolates Causing Dengue Outbreak, São Tomé and Príncipe, 2022

Appendix

Material and Methods

Sample collection and RNA extraction

Seven plasma samples were collected randomly from patients that had an RDT positive test at the Hospital Dr. Ayres Menezes between 6th and 16th of May 2022 (Appendix Table 2). RNA was extracted from plasma using the QIAamp Viral RNA Mini Kit (Qiagen #52906) according to the manufacturer's instructions. Aliquots of the extracted nucleic acid were stored at −80°C until further analyses.

Real-time PCR and serotype identification

To detect DENV and identify the serotype real-time PCR was carried out using the RealStar Dengue RT-PCR Kit 3.0 (Altona Diagnostics #283003) and the RealStar Dengue Type RT-PCR Kit 1.0 (Altona Diagnostics #621003) according to manufacturer's instructions on a 7500 real-time PCR Instrument (Applied Biosystems).

Oxford Nanopore Technologies (ONT) library preparation

cDNA synthesis and multiplex PCR amplification were performed as previously described (*1*). Because the subtyping by real-time PCR did not reveal any evidence of infections with other serotypes than DENV-3, we used two pools of serotype-specific multiplex primer sets specifically designed for whole genome sequencing (WGS) of DENV-3 (*1*). Following PCR amplification, the two reactions were pooled and the final steps of the library preparation for the portable MinION sequencer (Oxford Nanopore Technologies) were performed as described (*2*).

Whole genome sequencing and consensus genomes

The sequencing run was performed on a MinION M1kc device (Oxford Nanopore Technology) and conversion from raw data to nucleotide sequences ("basecalling") in high-accuracy mode was performed with ONT Guppy basecalling software version 6.5.7. Fastq files for each of the seven DENV-3 isolates were uploaded into the Genome Detective Platform for default viral analyses and assembly of the consensus sequence based on DENV-3 reference sequence NC_001475 using the Genome Detective Virus Tool v2.12.2 (https://www.genomedetective.com) (*3*).

Genotype identification

For genotype identification based on the consensus sequences of the seven DENV-3 virus isolates we used the web application Flavivirus Genotyping Tool v0.1 (https://www.rivm.nl/mpf/typingtool/flavivirus/) (*4*).

DENV-3 dataset and phylogenomic reconstruction

The alignment and subsequent reconstruction of phylogenomic relationships of all publicly available DENV-3 genotype III genomes together with the four DENV-3 from STP was carried out as described previously (*5*). Before the analysis, the four STP sequences were visually inspected. None of them contained any undetermined nucleotide in the consensus sequence. All DENV-3 genotype III genomes with a genome length >10,000bp that were available from GenBank via the NCBI Virus portal were downloaded for the phylogenomic analyses. The search on the assessment day (30–06–2023) resulted in a total of 1,168 DENV-3 genomes (Appendix Table 3). Hence, the complete dataset used for alignment with MAFFT v7.520 (*6*) included 1,172 DENV3 genomes. The reconstruction of the phylogenomic relationships was done by maximum likelihood (ML) using IQ-TREE 2 multicore v2.2.2.6 (*7*). The best-fitting evolutionary model was selected. A ML phylogenetic tree was constructed using the best-fitting model based on Bayesian information criterion (BIC) tests with ModelFinder (*8*) and Ultrafast bootstrap with 2,000 replicates (*9*). Visualization of the best fitting consensus tree was done in iTOLv6 (*10*).

References

1. Su W, Jiang L, Lu W, Xie H, Cao Y, Di B, et al. A serotype-specific and multiplex PCR method for whole-genome sequencing of dengue virus directly from clinical samples. Microbiol Spectr. 2022;10:e0121022. PubMed https://doi.org/10.1128/spectrum.01210-22

2. Quick J, Grubaugh ND, Pullan ST, Claro IM, Smith AD, Gangavarapu K, et al. Multiplex PCR method for MinION and Illumina sequencing of Zika and other virus genomes directly from clinical samples. Nat Protoc. 2017;12:1261–76. PubMed https://doi.org/10.1038/nprot.2017.066

3. Vilsker M, Moosa Y, Nooij S, Fonseca V, Ghysens Y, Dumon K, et al. Genome Detective: an automated system for virus identification from high-throughput sequencing data. Bioinformatics. 2019;35(5):871–3. **PMID 30124794**

4. National Institute for Public Health and the Environment. Flavivirus genotyping tool [cited 2023 Jul 18]. https://www.rivm.nl/mpf/typingtool/flavivirus

5. Naveca FG, Santiago GA, Maito RM, Ribeiro Meneses CA, do Nascimento VA, de Souza VC, et al. Reemergence of dengue virus serotype 3, Brazil, 2023. Emerg Infect Dis. 2023;29:1482–4. PubMed https://doi.org/10.3201/eid2907.230595

6. Katoh K, Standley DM. MAFFT multiple sequence alignment software version 7: improvements in performance and usability. Mol Biol Evol. 2013;30:772–80. PubMed https://doi.org/10.1093/molbev/mst010

7. Minh BQ, Schmidt HA, Chernomor O, Schrempf D, Woodhams MD, von Haeseler A, et al. IQ-TREE 2: new models and efficient methods for phylogenetic inference in the genomic era. Mol Biol Evol. 2020;37:1530–4. PubMed https://doi.org/10.1093/molbev/msaa015

8. Kalyaanamoorthy S, Minh BQ, Wong TKF, von Haeseler A, Jermiin LS. ModelFinder: fast model selection for accurate phylogenetic estimates. Nat Methods. 2017;14:587–9. PubMed https://doi.org/10.1038/nmeth.4285

9. Hoang DT, Chernomor O, von Haeseler A, Minh BQ, Vinh LS. UFBoot2: Improving the ultrafast bootstrap approximation. Mol Biol Evol. 2018;35:518–22. PubMed https://doi.org/10.1093/molbev/msx281

10. Letunic I, Bork P. Interactive Tree Of Life (iTOL) v5: an online tool for phylogenetic tree display and annotation. Nucleic Acids Res. 2021;49(W1):W293–6. PubMed https://doi.org/10.1093/nar/gkab301

Appendix Table 1. Age and sex distribution of 144 RDT-positive Dengue cases admitted to hospital during the 2022 Dengue outbreak in the Democratic Republic of São Tomé and Príncipe

Age interval [years]	No. of cases	Percentage [%]	Gender [M/F]
0–9	17	11.8	9/8
10–19	26	18.1	19/7
20–29	26	18.1	15/11
30–39	18	12.5	9/9
40–49	26	18.1	9/17
50–59	13	9.0	6/7
>60	18	12.5	5/13
Total	144	100.0	72/72

Appendix Table 2. Real-time PCR results for seven analyzed DENV-3 GIII isolates from the 2022 Dengue outbreak in the Democratic Republic of São Tomé and Príncipe*

ID	Collection date	Age [years]/ Gender	District	Ct	Serotype	Genotype
DENVSTP01	16.05.22	24/M	Água-Grande	24.9	3	III
DENVSTP02	06.05.22	40/M	Água-Grande	18.9	3	III
DENVSTP03	06.05.22	17/F	Água-Grande	28.3	3	III
DENVSTP04	12.05.22	8/F	Mezóchi	20.6	3	III
DENVSTP05	16.05.22	34/F	Água-Grande	20.6	3	III
DENVSTP06	16.05.22	25/F	Água-Grande	18.4	3	III
DENVSTP07	16.05.22	43/F	Água-Grande	16.1	3	III

*Ct, value of cycle threshold from real-time PCR

Appendix Table 3. Assembly statistics for seven sequenced DENV-3 GIII isolates from the 2022 Dengue outbreak in the Democratic Republic of São Tomé and Príncipe*

Sample ID	Ct value	#Reads	Coverage depth	NT Identity [%]	AA Identity [%]	Genome Coverage [%]	#Contigs	Genome length	ENA Accession number
DENVSTP01	24.9	3,460	169	95.7	98.5	91.5	3	9,798	–
DENVSTP02	18.9	51,915	2,302	95.7	98.4	97.9	1	10,484	ERS16303457
DENVSTP03	28.3	48	4	94.6	quality too low	45.9	10	4,911	–
DENVSTP04	20.6	1,108	71	95.6	98.3	66.2	8	7,083	–
DENVSTP05	20.6	5,166	259	95.7	98.6	97.7	1	10,461	ERS16303458
DENVSTP06	18.4	24,339	1,288	95.7	98.6	97.7	1	10,460	ERS16303459
DENVSTP07	16.1	64,440	4,148	95.8	98.7	98.3	1	10,526	ERS16303460

*The four in bold highlighted isolates were included in the phylogenomic analysis. Ct, real-time PCR cycle threshold; NT, nucleotide; AA, amino acid; Reference sequence used for assembly: NC_001475; ENA, European Nucleotide Archive.

Appendix Table 4. Publicly available DENV-3 GIII genomes used in the phylogenomic analysis (assessed on 30–06–2023)

Accession	Country	Collection date
AY099336	Sri Lanka	2000
AY099337	Martinique	1999
AY662691	Singapore	2004
AY679147	Brazil	2002
AY770511	India	2003
DQ675533	Taiwan	1999
EF629366	Brazil	2004–11
EF629367	Brazil	2004–11
EF629368	Brazil	2004–11
EF629369	Brazil	2002–01
EF643017	Brazil	2003
EU081181	Singapore	2004
EU081182	Singapore	2005
EU081183	Singapore	2005
EU081184	Singapore	2005
EU081185	Singapore	2005
EU081186	Singapore	2005
EU081187	Singapore	2005
EU081188	Singapore	2005
EU081189	Singapore	2005
EU081190	Singapore	2005
EU081191	Singapore	2005
EU081192	Singapore	2005
EU081193	Singapore	2005
EU081194	Singapore	2005
EU081195	Singapore	2005
EU081196	Singapore	2005
EU081197	Singapore	2005
EU081198	Singapore	2005
EU081199	Singapore	2005
EU081200	Singapore	2005
EU081201	Singapore	2005
EU081202	Singapore	2005
EU081203	Singapore	2005
EU081204	Singapore	2005
EU081205	Singapore	2005
EU081206	Singapore	2005
EU081207	Singapore	2005
EU081208	Singapore	2005
EU081209	Singapore	2005
EU081210	Singapore	2005
EU081211	Singapore	2005
EU081212	Singapore	2005
EU081213	Singapore	2005
EU081214	Singapore	2005
EU081215	Singapore	2005
EU081216	Singapore	2005
EU081217	Singapore	2005
EU081218	Singapore	2005
EU081219	Singapore	2005
EU081220	Singapore	2005
EU081222	Singapore	2005
EU081224	Singapore	2005
EU081225	Singapore	2005
EU482555	USA	2006
EU482558	USA	1998
EU482559	USA	1998
EU482563	USA	1998
EU482564	USA	2003
EU482566	USA	1998
EU482595	USA	2003
EU482596	USA	1998
EU482612	Venezuela	2001
EU482613	Venezuela	2001
EU482614	Venezuela	2001
EU529683	Venezuela	2007

Accession	Country	Collection date
EU529684	Venezuela	2001
EU529685	Venezuela	2001
EU529686	Venezuela	2001
EU529687	Venezuela	2001
EU529688	Venezuela	2001
EU529689	Venezuela	2001
EU529690	Venezuela	2001
EU529691	Venezuela	2001
EU529692	USA	2006
EU529696	USA	1999
EU529697	USA	2000
EU529698	USA	2006
EU529699	USA	2006
EU529702	USA	2003
EU529703	USA	1998
EU529704	USA	2004
EU529705	USA	2004
EU569688	Venezuela	2001
EU569689	Venezuela	2001
EU569690	Venezuela	2001
EU569691	Venezuela	2001
EU596492	USA	2007
EU596493	USA	2007
EU596494	USA	2007
EU660420	Venezuela	2001
EU687196	USA	2002
EU687197	USA	2003
EU687198	USA	2003
EU687218	USA	1998
EU687219	USA	1999
EU687221	USA	2000
EU687226	USA	1999
EU687233	USA	2002
EU687234	USA	2002
EU687239	USA	2003
EU726768	USA	2000
EU726769	USA	2003
EU726771	USA	1998
EU726772	USA	1998
EU726773	USA	1999
EU726774	USA	1999
EU781136	USA	1999
EU781137	USA	1999
EU854291	Venezuela	2004
EU854292	Venezuela	2005
EU854298	USA	2002
EU932687	Venezuela	2007
EU932688	Venezuela	2007
FJ024465	USA	2004
FJ024466	USA	2004
FJ024467	USA	2004
FJ024468	USA	2004
FJ024469	USA	2004
FJ024470	USA	2004
FJ024471	USA	2004
FJ177308	Brazil	2001
FJ182004	USA	2004
FJ182005	USA	2004
FJ182006	USA	2004
FJ182007	USA	2005
FJ182008	USA	2005
FJ182009	USA	2005
FJ182010	USA	2005
FJ182011	USA	2005
FJ182013	USA	1998
FJ182015	Venezuela	2001
FJ182037	USA	2005
FJ182038	USA	2005
FJ182039	USA	2005

Accession	Country	Collection date
FJ182040	USA	2005
FJ182041	USA	2005
FJ205870	USA	2003
FJ205871	USA	1999
FJ373302	USA	2004
FJ373303	Venezuela	2001
FJ373304	Venezuela	2004
FJ373306	USA	2002
FJ390371	USA	2003
FJ390372	USA	2003
FJ390373	USA	2002
FJ390375	USA	1999
FJ390376	USA	1999
FJ390377	USA	1999
FJ410176	USA	2000
FJ410177	USA	2000
FJ410178	USA	2002
FJ478456	USA	2002
FJ547069	USA	1999
FJ547070	USA	1998
FJ547071	USA	2000
FJ547072	USA	2000
FJ547073	USA	2000
FJ547074	USA	2000
FJ547075	USA	2000
FJ547076	USA	2000
FJ547077	USA	2000
FJ547078	USA	2000
FJ547079	USA	2001
FJ547080	USA	2001
FJ547081	USA	2001
FJ547082	USA	2001
FJ547083	USA	2002
FJ547084	USA	2002
FJ547085	USA	2006
FJ562107	USA	2000
FJ639746	Venezuela	2000
FJ639747	Venezuela	2000
FJ639749	Venezuela	2000
FJ639750	Venezuela	2000
FJ639751	Venezuela	2001
FJ639752	Venezuela	2001
FJ639753	Venezuela	2001
FJ639754	Venezuela	2001
FJ639755	Venezuela	2001
FJ639756	Venezuela	2001
FJ639757	Venezuela	2001
FJ639758	Venezuela	2001
FJ639759	Venezuela	2001
FJ639760	Venezuela	2001
FJ639761	Venezuela	2001
FJ639762	Venezuela	2001
FJ639763	Venezuela	2001
FJ639765	Venezuela	2001
FJ639766	Venezuela	2001
FJ639767	Venezuela	2001
FJ639768	Venezuela	2001
FJ639769	Venezuela	2001
FJ639770	Venezuela	2001
FJ639771	Venezuela	2001
FJ639772	Venezuela	2007
FJ639774	Venezuela	2001
FJ639775	Venezuela	2002
FJ639776	Venezuela	2002
FJ639777	Venezuela	2002
FJ639778	Venezuela	2002
FJ639779	Venezuela	2003
FJ639780	Venezuela	2003
FJ639781	Venezuela	2003

Accession	Country	Collection date
FJ639782	Venezuela	2003
FJ639784	Venezuela	2003
FJ639785	Venezuela	2003
FJ639786	Venezuela	2003
FJ639787	Venezuela	2004
FJ639789	Venezuela	2004
FJ639790	Venezuela	2004
FJ639791	Venezuela	2004
FJ639792	Venezuela	2004
FJ639793	Venezuela	2004
FJ639795	Venezuela	2004
FJ639798	Venezuela	2004
FJ639799	Venezuela	2004
FJ639800	Venezuela	2004
FJ639801	Venezuela	2004
FJ639803	Venezuela	2005
FJ639804	Venezuela	2005
FJ639805	Venezuela	2005
FJ639807	Venezuela	2005
FJ639810	Venezuela	2005
FJ639816	Venezuela	2005
FJ639817	Venezuela	2006
FJ639825	Venezuela	2006
FJ639826	Venezuela	2008
FJ639827	Venezuela	2008
FJ644564	India	2007
FJ744700	Venezuela	2001
FJ810416	Venezuela	2001
FJ850048	Nicaragua	2008
FJ850049	Nicaragua	2008
FJ850052	Nicaragua	2008
FJ850055	USA	2004
FJ850056	USA	2004
FJ850079	Brazil	2003
FJ850080	Brazil	2003
FJ850083	Brazil	2004
FJ850086	Brazil	2005
FJ850089	Brazil	2006
FJ850092	Brazil	2007
FJ850094	Brazil	2008
FJ850096	Venezuela	2001
FJ850097	Venezuela	2001
FJ850098	Venezuela	2001
FJ850109	Venezuela	2007
FJ850110	Venezuela	2007
FJ850111	Venezuela	2007
FJ873812	Nicaragua	2008
FJ873813	Nicaragua	2008
FJ882571	Sri Lanka	1989
FJ882572	Sri Lanka	1989
FJ882573	Sri Lanka	1993
FJ882574	Sri Lanka	1985
FJ882575	Mozambique	1985
FJ882576	Nicaragua	1994
FJ882577	Venezuela	2001
FJ882578	Venezuela	2001
FJ898440	Mexico	2003
FJ898441	Mexico	2006
FJ898442	Mexico	2007
FJ898443	Colombia	2003
FJ898444	Colombia	2005
FJ898445	Colombia	2007
FJ898446	Brazil	2001
FJ898447	Brazil	2003
FJ898457	Ecuador	2000
FJ898458	Peru	2002
FJ898459	Trinidad and Tobago	2002
FJ898462	Anguilla	2001
FJ898463	Saint Lucia	2001

Accession	Country	Collection date
FJ898464	Guyana	2002
FJ898468	Venezuela	2000
FJ898469	Venezuela	2001
FJ898470	Venezuela	2001
FJ898471	Venezuela	2002
FJ898472	Venezuela	2003
FJ898473	Venezuela	2003
FJ898474	Venezuela	2007
FJ898475	Nicaragua	2008
FJ898476	Nicaragua	2008
FJ913015	Brazil	2001
GQ199860	Nicaragua	2008
GQ199861	Nicaragua	2008
GQ199862	Nicaragua	2008
GQ199863	Nicaragua	2008
GQ199864	Nicaragua	2008
GQ199865	Nicaragua	2009
GQ199870	Nicaragua	2008
GQ199871	Nicaragua	2008
GQ199886	Nicaragua	1998
GQ199887	Sri Lanka	1983
GQ199888	Sri Lanka	1983
GQ199889	Sri Lanka	1983
GQ199891	Colombia	2001
GQ252674	Sri Lanka	1997
GQ252678	Venezuela	2001
GQ466079	India	2008
GQ868546	Brazil	2006
GQ868547	Brazil	2006
GQ868548	Brazil	2006
GQ868571	Colombia	2002
GQ868572	Colombia	2003
GQ868573	Colombia	2003
GQ868574	Colombia	2003
GQ868575	Colombia	2004
GQ868576	Colombia	2005
GQ868577	Colombia	2005
GQ868578	Colombia	2007
GQ868586	Venezuela	2007
GQ868587	Venezuela	2007
GQ868616	Saint Lucia	2001
GQ868617	Trinidad and Tobago	2002
GU131844	Brazil	2006
GU131845	Brazil	2006
GU131846	Brazil	2006
GU131847	Brazil	2006
GU131848	Brazil	2006
GU131849	Brazil	2006
GU131850	Brazil	2006
GU131851	Brazil	2006
GU131852	Brazil	2006
GU131853	Brazil	2006
GU131854	Brazil	2006
GU131855	Brazil	2006
GU131856	Brazil	2006
GU131857	Brazil	2006
GU131858	Brazil	2006
GU131859	Brazil	2006
GU131860	Brazil	2006
GU131861	Brazil	2007
GU131862	Brazil	2007
GU131865	Brazil	2006
GU131866	Brazil	2007
GU131867	Brazil	2007
GU131868	Brazil	2007
GU131869	Brazil	2007
GU131870	Brazil	2007
GU131871	Brazil	2007
GU131872	Brazil	2007

Accession	Country	Collection date
GU131873	Brazil	2007
GU131874	Brazil	2007
GU131875	Brazil	2007
GU131876	Brazil	2007
GU131877	Brazil	2007
GU131878	Brazil	2007
GU131950	Colombia	2001
GU131951	Colombia	2003
GU131952	Colombia	2003
GU131953	Colombia	2004
GU131954	Colombia	2006
GU363549	China	2009–08–06
GU370053	Singapore	2007–05
HM181972	Nicaragua	2009
HM181973	Nicaragua	2009
HM181974	Nicaragua	2009
HM181975	Nicaragua	2009
HM181976	Nicaragua	2009
HM181977	Nicaragua	2009
HM181978	Nicaragua	2009
HM631856	Nicaragua	2009
HM631857	Nicaragua	2009
HM631858	Nicaragua	2009
HM631859	Nicaragua	2009
HM631860	Nicaragua	2009
HM631861	Nicaragua	2009
HM631862	Nicaragua	2009
HM631863	Nicaragua	2009
HM631864	Nicaragua	2009
HM631869	Nicaragua	2009
HM756274	Nicaragua	2009
HM756275	Nicaragua	2009
HM756276	Nicaragua	2009
HM756277	Nicaragua	2009
HM756278	Nicaragua	2009
HM756279	Nicaragua	2009
HM756280	Nicaragua	2009
HM756281	Nicaragua	2009
HM756282	Nicaragua	2009
HQ166030	Nicaragua	2009
HQ166031	Nicaragua	2009
HQ166032	Nicaragua	2009
HQ166033	Nicaragua	2009
HQ166034	Nicaragua	2009
HQ235027	Paraguay	2007
HQ332170	Venezuela	2006
HQ332171	Venezuela	2006
HQ541785	Nicaragua	2009
HQ541789	Nicaragua	2008
HQ541790	Nicaragua	2008
HQ541791	Nicaragua	2008
HQ541795	Nicaragua	2009
HQ541796	Nicaragua	2009
HQ541797	Nicaragua	2009
HQ541802	Nicaragua	2009
HQ541804	Nicaragua	2009
HQ541806	Nicaragua	2009
HQ671176	Nicaragua	2009
HQ671177	Nicaragua	2009
HQ671178	Nicaragua	2009
HQ705609	Nicaragua	2009
HQ705610	Nicaragua	2009
HQ705611	Nicaragua	2009
HQ705612	Nicaragua	2009
HQ705613	Nicaragua	2009
HQ705614	Nicaragua	2009
HQ705615	Nicaragua	2009
HQ705616	Nicaragua	2009
HQ705617	Nicaragua	2009

Accession	Country	Collection date
HQ705618	Nicaragua	2009
HQ705619	Nicaragua	2009
HQ705620	Nicaragua	2009
HQ705621	Nicaragua	2009
HQ705622	Nicaragua	2009
HQ705623	Nicaragua	2009
HQ891025	Nicaragua	2009
JF504679	China	2009–09
JF808118	Brazil	2002
JF808119	Brazil	2004
JF808120	Brazil	2009
JF808121	Brazil	2007
JF808122	Paraguay	2003
JF808123	Paraguay	2002
JF808124	Brazil	2003
JF808125	Brazil	2002
JF808126	Brazil	2003
JF808127	Brazil	2002
JF808128	Paraguay	2003
JF808129	Paraguay	2003
JF920393	Nicaragua	2009
JF920394	Nicaragua	2009
JF920395	Nicaragua	2009
JF920396	Nicaragua	2009
JF920397	Nicaragua	2010
JF920398	Nicaragua	2009
JF920399	Nicaragua	2009
JF920400	Nicaragua	2009
JF920401	Nicaragua	2010
JF920402	Nicaragua	2009
JF920403	Nicaragua	2009
JF920404	Nicaragua	2009
JF920405	Nicaragua	2010
JF920406	Nicaragua	2010
JF920407	Nicaragua	2009
JF920408	Nicaragua	2010
JF920409	Nicaragua	2010
JF937620	Nicaragua	2010
JF937621	Nicaragua	2010
JF937622	Nicaragua	2010
JF937623	Nicaragua	2010
JF937624	Nicaragua	2010
JF937625	Nicaragua	2010
JF937626	Nicaragua	2010
JF937627	Nicaragua	2010
JF937628	Nicaragua	2010
JF937629	Nicaragua	2010
JF937630	Nicaragua	2009
JF937631	Nicaragua	2009
JF937632	Nicaragua	2009
JF937633	Nicaragua	2010
JF937634	Nicaragua	2010
JF937636	Nicaragua	2009
JF937637	Nicaragua	2010
JF937638	Nicaragua	2010
JF937639	Nicaragua	2010
JF937640	Nicaragua	2010
JF937641	Nicaragua	2009
JF937642	Nicaragua	2009
JF937643	Nicaragua	2009
JF937646	Nicaragua	2010
JF937647	Nicaragua	2009
JF937648	Nicaragua	2010
JF937652	Nicaragua	2009
JN000936	Nicaragua	2009
JN000937	Nicaragua	2010
JN000938	Nicaragua	2010
JN093513	Nicaragua	2010
JN093514	Nicaragua	2009

Accession	Country	Collection date
JN093515	Nicaragua	2010
JN093517	Nicaragua	2009
JN183884	Nicaragua	2009
JN662391	China	2009–08–06
JQ411814	Sri Lanka	1989
JQ922555	India	1966
JQ922556	India	2005
JQ922557	India	2005
JX669489	Brazil	2003
JX669490	Brazil	2002
JX669491	Brazil	2002
JX669492	Brazil	2005
JX669493	Brazil	2005
JX669494	Brazil	2005
JX669495	Brazil	2004
JX669496	Brazil	2006
JX669497	Brazil	2005
JX669498	Brazil	2004
JX669499	Brazil	2004
JX669500	Brazil	2005
JX669501	Brazil	2005
JX669502	Brazil	2005
JX669503	Brazil	2005
JX669504	Brazil	2006
JX669505	Brazil	2006
JX669506	Brazil	2006
JX669507	Brazil	2006
JX669508	Brazil	2006
KC425219	Brazil	2002
KF041254	Pakistan	2008
KF041255	Pakistan	2007
KF041256	Pakistan	2006
KF041257	Pakistan	2006
KF041258	Pakistan	2009
KF041259	Pakistan	2006
KF921913	Nicaragua	2009
KF921914	Nicaragua	2009
KF921916	Nicaragua	2009
KF921920	Nicaragua	2009
KF921921	Nicaragua	2009
KF921922	Nicaragua	2009
KF921923	Nicaragua	2009
KF921924	Nicaragua	2010
KF921926	Nicaragua	2010
KF921928	Nicaragua	2010
KF921929	Nicaragua	2010
KF954945	China	2013–08–08
KF954946	China	2013–08–08
KF954947	China	2013–08–08
KF954948	China	2013–08–08
KF954949	China	2013–08–08
KF955449	Venezuela	2001–10–13
KF955451	Venezuela	2003–12–01
KF955453	Venezuela	2003–12–19
KF955454	Venezuela	2004–11–07
KF955456	Puerto Rico	2006
KF955465	Puerto Rico	2000
KF955466	Puerto Rico	2000
KF955468	Puerto Rico	2001
KF955471	Venezuela	2004
KF955472	Venezuela	2004
KF955473	Brazil	2002
KF955474	Sri Lanka	1989
KF955479	Venezuela	2001
KF955481	Venezuela	2007
KF955486	Venezuela	2001
KF955487	Venezuela	2001
KF955490	Nicaragua	2008
KF955505	Grenada	2002

Accession	Country	Collection date
KF971695	Nicaragua	2009
KF971709	Nicaragua	2009
KF973476	Nicaragua	2011
KF973477	Nicaragua	2011
KF973478	Nicaragua	2012
KF973479	Nicaragua	2012
KF973480	Nicaragua	2012
KF973481	Nicaragua	2011
KF973482	Nicaragua	2011
KF973483	Nicaragua	2011
KF973484	Nicaragua	2011
KF973485	Nicaragua	2011
KF973486	Nicaragua	2012
KF973487	Nicaragua	2011
KJ189255	Peru	2002
KJ189256	Peru	2002
KJ189257	Peru	2004
KJ189258	Peru	2002
KJ189259	Peru	2002
KJ189260	Peru	2002
KJ189261	Peru	2008
KJ189262	Peru	2004
KJ189263	Peru	2004
KJ189264	Peru	2004
KJ189265	Peru	2004
KJ189266	Peru	2004
KJ189267	Peru	2007
KJ189268	Peru	2007
KJ189269	Peru	2007
KJ189270	Peru	2007
KJ189271	Peru	2007
KJ189272	Peru	2007
KJ189273	Peru	2007
KJ189274	Peru	2007
KJ189275	Peru	2007
KJ189276	Peru	2007
KJ189277	Peru	2007
KJ189278	Peru	2007
KJ189279	Peru	2007
KJ189280	Peru	2007
KJ189281	Peru	2007
KJ189282	Peru	2007
KJ189283	Peru	2007
KJ189284	Peru	2008
KJ189285	Peru	2008
KJ189286	Peru	2008
KJ189287	Peru	2008
KJ189288	Peru	2008
KJ189289	Peru	2008
KJ189290	Peru	2008
KJ189291	Peru	2008
KJ189292	Peru	2009
KJ189293	Peru	2005
KJ189294	Peru	2006
KJ189295	Peru	2006
KJ189296	Peru	2006
KJ189297	Peru	2006
KJ189298	Peru	2008
KJ189299	Peru	2005
KJ189300	Peru	2008
KJ189301	Peru	2008
KJ643590	Peru	2007
KJ830751	Saudi Arabia	2014–01–26
KT726340	Cuba	2001
KT726341	Cuba	2001
KT726342	Cuba	2001
KT726343	Cuba	2001
KT726344	Cuba	2001
KT726345	Cuba	2002

Accession	Country	Collection date
KT726346	Cuba	2002
KT726347	Cuba	2002
KT726348	Cuba	2002
KT726349	Cuba	2001
KT726350	Cuba	2001
KT726351	Cuba	2001
KT726352	Cuba	2001
KT726353	Cuba	2001
KT726354	Cuba	2001
KT726355	Cuba	2001
KT726356	Cuba	2001
KT726357	Cuba	2001
KT726358	Cuba	2001
KT726359	Cuba	2001
KT726360	Cuba	2001
KT726361	Cuba	2002
KU216208	India	2013–11–11
KU216209	India	2013–11–11
KU509278	Barbados	2007
KU509281	India	2009
KU509282	Senegal	2009
KU509283	Sri Lanka	2006
KU509286	India	2011
KX380841	Singapore	2012
KX380842	Singapore	2013
KX855927	India	2014–10–15
KY921907	Singapore	2015–04
LC379193	Gabon	2016–05–15
LC379194	Gabon	2016–05–20
LC379195	Gabon	2016–07–14
LC379196	Gabon	2016–07–22
LC379197	Gabon	2017–04–15
LC410192	Thailand	2016–10
LC410193	Thailand	2016–12
LC410194	Thailand	2016–12
LC410195	Thailand	2017–01
LT898451	Malaysia	2011
LT898452	Malaysia	2011
LT996904	Malaysia	2007
LT996905	Malaysia	2008
LT996906	Malaysia	2008
LT996907	Malaysia	2010
LT996908	Malaysia	2010
LT996909	Malaysia	2010
LT996910	Malaysia	2010
LT996911	Malaysia	2010
LT996912	Malaysia	1987
MF142763	Thailand	2015–09
MF370226	China	2013–08–20
MG721059	India	2016
MG721061	India	2016
MG721064	India	2016
MH048677	Malaysia	2014–12
MH051731	Malaysia	2014–12
MH051733	Malaysia	2014–12
MH544647	Colombia	2015–08–23
MH544649	Colombia	2015–09–15
MH544650	Colombia	2015–09–07
MH544651	Colombia	2016–04–16
MH822957	India	2013
MH888333	Bolivia	2011–01–01
MH891766	India	2017–02–21
MK858149	India	2016–08–26
MK858150	India	2016–08–10
MK858151	India	2016–11–08
MK858152	India	2016–08–24
MK858153	India	2016–10–14
MK858154	India	2017–10–12
MK858155	India	2017–10–09

Accession	Country	Collection date
MK894339	China	2018–04–09
MK894340	China	2018–10–16
MK894341	China	2018–12–30
MN018367	China	2015–09–13
MN018368	China	2013–07–25
MN018371	China	2015–08–08
MN018372	China	2016–03–18
MN018375	China	2015–10–23
MN018376	China	2015–07–06
MN018378	China	2015–09–30
MN018381	China	2016–05–06
MN018385	China	2016–09–20
MN018386	China	2013–07–28
MN227697	China	2019–05–19
MN227698	China	2019–07–02
MN227699	China	2019–06–30
MN227700	China	2019–07–08
MN227701	China	2019–07–08
MN227702	China	2019–06–01
MN227703	China	2019–07–18
MN253124	India	2017–08–10
MN253125	India	2017–10–27
MN253126	India	2016
MN253127	India	2018–09–06
MN253128	India	2018–09–18
MN253129	India	2018–09–20
MN253130	India	2018–12–09
MN253131	India	2018–12–09
MN253132	India	2018–09–18
MN253133	India	2018–10–13
MN448966	Thailand	2012–09–25
MN448967	Thailand	2012–09–25
MN448986	Thailand	2011–06–14
MN448991	Thailand	2012–05–11
MN448992	Thailand	2012–10–01
MN453624	Singapore	2016–01–20
MN922036	China	2019–07–17
MN964273	China	2019–11–09
MN964274	China	2019–11–21
MT261972	Burkina Faso	2017–10–16
MT261973	Burkina Faso	2017–11–09
MT261974	Burkina Faso	2017–11–13
MT261975	Burkina Faso	2017–11–17
MT261976	Burkina Faso	2017–10–04
MT261977	Burkina Faso	2017–10–12
MT261978	Burkina Faso	2017–10–27
MT261979	Burkina Faso	2017–11–02
MW192820	India	2016–08
MW192821	India	2016–08
MW192822	India	2016–08
MW192823	India	2016–07
MW192824	India	2017–08
MW288025	Senegal	2018–10
MW288026	Senegal	2018–10
MW288027	Senegal	2018–10
MW288028	Senegal	2018–10
MW288031	Senegal	2018–11
MW288033	Senegal	2018–11
MW288035	Senegal	2018–11
MW288037	Senegal	2018–11
MW288038	Senegal	2018–11
MW288039	Senegal	2018–11
MW288040	Senegal	2018–11
MW720883	China	2019–09
MW720884	China	2019–09
MW720885	China	2019–09
MW720886	China	2019–09
MW720887	China	2019–09
MW720888	China	2019–09

Accession	Country	Collection date
MW788883	Myanmar	2017–06
MW788884	Myanmar	2017–07
MW788886	Myanmar	2017–07
MW788887	Myanmar	2017–07
MW788888	Myanmar	2017–07
MW788889	Myanmar	2018–07
MW788891	Myanmar	2019–12
MW788893	Myanmar	2019–07
MW788896	Myanmar	2019–05
MW788904	Myanmar	2018–07
MW788906	Myanmar	2018–06
MW788907	Myanmar	2018–10
MW788910	Myanmar	2018–08
MW788911	Myanmar	2018–07
MW945428	Puerto Rico	2006
MW946612	Thailand	2010
MW946615	Thailand	2014
MW946633	Thailand	2013
MW946659	Thailand	2013
MW946662	Thailand	2013
MW946679	Thailand	2014
MW946690	Thailand	2014
MW946693	Thailand	2012
MW946694	Thailand	2014
MW946704	Thailand	2013
MW946711	Thailand	2012
MW946721	Thailand	2011
MW946741	Thailand	2014
MW946763	Thailand	2013
MW946767	Thailand	2006
MW946775	Thailand	2014
MW946778	Thailand	2013
MW946783	Thailand	2012
MW946798	Thailand	2013
MW946799	Thailand	2012
MW946800	Thailand	2012
MW946801	Thailand	2014
MW946802	Thailand	2014
MW946808	Thailand	2010
MW946810	Thailand	2012
MW946815	Thailand	2006
MW946839	Thailand	2011
MW946841	Thailand	2012
MW946843	Thailand	2014
MW946870	Thailand	2014
MW946872	Thailand	2014
MW946881	Thailand	2014
MW946891	Thailand	2011
MW946918	Nicaragua	2009
MW946957	Thailand	2013
MW946958	Thailand	2002
MW946973	Thailand	2013
MW946974	Thailand	2011
MW946979	Thailand	2014
MZ008468	Nicaragua	2014
MZ008469	Nicaragua	2013
MZ008470	Nicaragua	2013
MZ008471	Nicaragua	2013
MZ008472	Nicaragua	2013
MZ008473	Nicaragua	2013
MZ008474	Nicaragua	2013
MZ008475	Nicaragua	2013
MZ008476	Nicaragua	2014
MZ008477	Nicaragua	2013
MZ008478	Nicaragua	2013
MZ312921	India	2018–07–09
MZ544585	Kenya	2019–03
MZ544586	Kenya	2019–03
MZ544587	Kenya	2019–03

Accession	Country	Collection date
MZ544588	Kenya	2019–03
MZ857204	Kenya	2011
MZ857217	Saudi Arabia	2016
OK605762	Paraguay	2007
OK605765	Ecuador	2001
OK605766	Somalia	1993
OM368353	China	2019–05–17
OM417340	Mexico	2021–09–21
OM417341	Mexico	2021–09–19
OM638675	India	2021–11–29
OM865777	Bhutan	2019–07–29
OM865778	Bhutan	2019–07–29
OM865779	Bhutan	2019–07–29
OM865780	Bhutan	2019–07–30
OM865781	Bhutan	2019–07–31
OM865782	Bhutan	2019–08–03
OM865783	Bhutan	2019–08–02
OM865784	Bhutan	2019–08–08
OM865785	Bhutan	2019–08–08
OM865786	Bhutan	2019–08–03
OM865787	Bhutan	2019–08–06
OM865788	Bhutan	2019–08–09
OM865789	Bhutan	2019–08–07
OM865790	Bhutan	2019–08–09
OM865791	Bhutan	2019–08–10
OM865792	Bhutan	2019–08
OM865793	Bhutan	2019–07–19
OM865794	Bhutan	2019–07–27
OM865795	Bhutan	2019–08–24
OM865796	Bhutan	2019–08–20
OM865797	Bhutan	2019–08–20
OM865798	Bhutan	2019–08–22
OM865799	Bhutan	2019–08–19
OM865800	Bhutan	2019–08–19
OM865801	Bhutan	2019–08–19
OM865802	Bhutan	2019–07–17
OM865803	Bhutan	2019–07–17
OM865804	Bhutan	2019–07–28
OM865805	Bhutan	2019–07–18
OM865806	Bhutan	2019–08–26
OM865807	Bhutan	2019–08–24
OM865808	Bhutan	2019–08–28
OM865809	Bhutan	2019–09–04
OM865810	Bhutan	2019–09–02
OM865811	Bhutan	2019–08–31
OM865812	Bhutan	2019–09–07
OM865813	Bhutan	2019–09–09
OM865814	Bhutan	2019–09–09
OM865815	Bhutan	2019–09–10
OM865816	Bhutan	2019–09–14
OM865817	Bhutan	2019–09–21
OM865818	Bhutan	2019–08
OM865819	Bhutan	2019–08
OM865820	Bhutan	2019–08
ON007080	Thailand	2011–08–04
ON055565	Thailand	2010–02–16
ON055567	Thailand	2011–08–25
ON109599	India	2021
ON123655	India	2020
ON123658	India	2019
ON123659	India	2018
ON123660	India	2018
ON123662	India	2018
ON123665	India	2018
ON123669	India	2018
ON123670	India	2018
ON799401	India	2018
ON890788	Ethiopia	2019–11–09
ON890789	Maldives	2019–05–19

Accession	Country	Collection date
ON890819	China	2019–07–08
ON891145	China	2019–06–01
ON900159	China	2019–06–01
ON907582	China	2019–07–08
ON908232	Ethiopia	2019–11–09
ON908233	Maldives	2019–05–19
ON908234	China	2019–07–08
ON908245	China	2019–06–01
OP410993	Singapore	2008–09
OP410994	Singapore	2013–01
OP410997	Singapore	2019–01
OP410998	Singapore	2018–02
OP895705	India	2018
OP895928	Maldives	2019–03
OP895929	Maldives	2019–12
OP921002	India	2022–07–27
OQ339138	India	2022
OQ445895	Cuba	2022–08–01
OQ445896	Cuba	2022–07–25
OQ445897	Cuba	2022–07–25
OQ445898	Cuba	2022–07–30
OQ445899	Cuba	2022–07–28
OQ445901	Cuba	2022–07–25
OQ445902	Cuba	2022–07–26
OQ445903	Cuba	2022–07–19
OQ445904	Cuba	2022–07–30
OQ445905	Cuba	2022–08–02
OQ445906	Cuba	2022–08–03
OQ445907	Cuba	2022–08–03
OQ445908	Cuba	2022–07–27
OQ445909	Cuba	2022–08–08
OQ445910	Cuba	2022–08–08
OQ445911	Cuba	2022–08–06
OQ445912	Cuba	2022–08–09
OQ445913	Cuba	2022–08–09
OQ445914	Cuba	2022–08–04
OQ445915	Cuba	2022–08–10
OQ445916	Cuba	2022–08–10
OQ445917	Cuba	2022–08–08
OQ445918	USA	2022–08–09
OQ445919	USA	2022–08–15
OQ445920	USA	2022–08–18
OQ445921	USA	2022–08–28
OQ445922	USA	2022–08–29
OQ445923	USA	2022–09–05
OQ445924	Cuba	2022–09–02
OQ445925	USA	2022–09–14
OQ445926	Cuba	2022–09–11
OQ445927	USA	2022–09–25
OQ445928	Cuba	2022–09–20
OQ445929	USA	2022–09–25
OQ445930	USA	2022–10–10
OQ445931	USA	2022–10–19
OQ445932	USA	2022–10–23
OQ445933	USA	2022–10–24
OQ445934	USA	2022–10–20
OQ445935	USA	2022–10–26
OQ445936	Cuba	2022–07–26
OQ445937	Cuba	2022–07–22
OQ445938	Cuba	2022–07–21
OQ445939	Cuba	2022–07–22
OQ445940	Cuba	2022–07–09
OQ445941	Cuba	2022–07–21
OQ445942	Cuba	2022–07–22
OQ445943	Cuba	2022–07–19
OQ445944	Cuba	2022–07–22
OQ445945	Cuba	2022–07–17
OQ445946	USA	2022–08–09
OQ445947	USA	2022–08–05

Accession	Country	Collection date
OQ445948	USA	2022–07–29
OQ445949	Cuba	2022–07–15
OQ445950	Cuba	2022–07–09
OQ445951	Cuba	2022–07–09
OQ445952	Cuba	2022–07–10
OQ445953	USA	2022–07–13
OQ445954	Cuba	2022–07–08
OQ445955	Cuba	2022–07–09
OQ445956	Cuba	2022–07–06
OQ445957	Cuba	2022–06–25
OQ445958	Cuba	2022–06–28
OQ445959	Cuba	2022–06–29
OQ445960	Cuba	2022–06–21
OQ445961	Cuba	2022–05–22
OQ445962	Cuba	2022–06–28
OQ836205	Puerto Rico	2022–10–29
OQ836206	Puerto Rico	2022–08–20
OQ836207	Puerto Rico	2022–09–02
OQ836208	Puerto Rico	2022–12–27
OQ836209	Puerto Rico	2023–01–24
OQ836210	Puerto Rico	2022–07–30
OQ836211	Puerto Rico	2022–07–27
OQ727062	Brazil	2023–03–12
OQ868517	Brazil	2023–03–12
OQ706226	Brazil	2023–03–04
OQ706227	Brazil	2023–01–22
OQ706228	Brazil	2023–01–03
OR150744	USA	19.12.22
OR150745	USA	22.12.22
OR150746	USA	18.12.22
OR150747	USA	23.12.22
OR150748	USA	01.01.23
OR150749	USA	06.01.23
OR150750	USA	10.01.23
OR150751	USA	09.01.23
OR150752	USA	11.01.23
OR150753	USA	03.01.23
OR150754	USA	08.01.23
OR150755	USA	08.01.23
OR162311	USA	27.11.22
OR162312	USA	02.12.22
OR162313	USA	27.11.22
OR162314	USA	04.12.22
OR162315	USA	06.12.22
OR162316	USA	02.12.22
OR162317	USA	04.12.22
OR162318	USA	10.12.22
OR162319	USA	18.12.22
OQ948473	China	2019–09
OQ948474	China	2019–09
OQ603303	Colombia	17.08.15
OQ603304	Colombia	30.06.15
OQ603305	Colombia	27.08.15
OQ603306	Colombia	21.08.15
OQ603307	Colombia	2015
OQ603308	Colombia	05.09.15
OQ603309	Colombia	15.09.15
OQ603310	Colombia	2015
OQ603311	Colombia	2015
OQ603312	Colombia	21.01.16
OQ603313	Colombia	26.04.16
OQ603314	Colombia	20.07.16
OQ603315	Colombia	05.08.16
OR029719	China	27.07.19
OQ132878	Niger	18.08.22
OQ919689	USA	22.09.22
OQ919690	Cuba	05.10.22
OQ919691	USA	08.11.22
OQ919692	Cuba	04.12.22

Accession	Country	Collection date
OQ919693	Cuba	20.08.22
OQ857505	USA	01.09.22
OQ857506	USA	05.10.22
OQ857507	USA	07.11.22
OQ821502	Colombia	12.08.21
OQ821503	Cuba	13.01.16
OQ821504	Cuba	06.07.22
OQ821505	Cuba	17.07.22
OQ821506	Cuba	23.07.22
OQ821507	Cuba	28.07.22
OQ821508	Cuba	28.07.22
OQ821509	Cuba	05.08.22
OQ821510	Cuba	06.08.22
OQ821511	Cuba	09.08.22
OQ821512	Cuba	10.08.22
OQ821513	Cuba	12.08.22
OQ821514	Cuba	13.08.22
OQ821515	Cuba	14.08.22
OQ821516	Cuba	15.08.22
OQ821517	Cuba	16.08.22
OQ821518	Cuba	18.08.22
OQ821519	Cuba	18.08.22
OQ821520	Cuba	18.08.22
OQ821521	Cuba	18.08.22
OQ821522	Cuba	18.08.22
OQ821523	Cuba	18.08.22
OQ821524	Cuba	20.08.22
OQ821525	Cuba	21.08.22
OQ821526	Cuba	23.08.22
OQ821527	Cuba	23.08.22
OQ821528	Cuba	24.08.22
OQ821529	Cuba	24.08.22
OQ821530	Cuba	24.08.22
OQ821531	Cuba	28.08.22
OQ821532	Cuba	30.08.22
OQ821533	Cuba	31.08.22
OQ821534	Cuba	31.08.22
OQ821535	Cuba	01.09.22
OQ821536	Cuba	03.09.22
OQ821537	Cuba	05.09.22
OQ821538	Cuba	05.09.22
OQ821539	Cuba	07.09.22
OQ821540	Cuba	08.09.22
OQ821541	Cuba	12.09.22
OQ821542	Cuba	13.09.22
OQ821543	Cuba	13.09.22
OQ821544	Cuba	14.09.22
OQ821545	Cuba	15.09.22
OQ821546	Cuba	17.09.22
OQ821547	Cuba	17.09.22
OQ821548	Cuba	21.09.22
OQ821549	Cuba	22.09.22
OQ821550	Cuba	22.09.22
OQ821551	Cuba	23.09.22
OQ821552	Cuba	23.09.22
OQ821553	Cuba	23.09.22
OQ821554	Cuba	25.09.22
OQ821555	Cuba	25.09.22
OQ821556	Cuba	25.09.22
OQ821557	Cuba	26.09.22
OQ821558	Cuba	26.09.22
OQ821559	Cuba	29.09.22
OQ821560	Cuba	29.09.22
OQ821561	Cuba	30.09.22
OQ821562	Cuba	02.10.22
OQ821563	Cuba	05.10.22
OQ821564	Cuba	07.10.22
OQ821565	Cuba	14.10.22
OQ821566	Cuba	15.10.22

Accession	Country	Collection date
OQ821567	Cuba	19.10.22
OQ821568	Cuba	20.10.22
OQ821569	Cuba	21.10.22
OQ821570	Cuba	30.10.22
OQ821571	Cuba	03.11.22
OQ821572	Cuba	03.11.22
OQ821573	Cuba	04.11.22
OQ821574	Cuba	07.11.22
OQ821575	Cuba	10.11.22
OQ821576	Cuba	14.11.22
OQ821577	Cuba	18.11.22
OQ821578	Cuba	18.11.22
OQ821579	Cuba	20.11.22
OQ821580	Cuba	25.11.22
OQ821581	Cuba	29.11.22
OQ821582	Cuba	30.11.22
OQ821583	Cuba	30.11.22
OQ821584	Cuba	30.11.22
OQ821585	Cuba	02.12.22
OQ821586	Cuba	04.12.22
OQ821587	Cuba	04.12.22
OQ821588	Cuba	06.12.22
OQ821589	Cuba	06.12.22
OQ821590	Cuba	08.12.22
OQ821591	Cuba	15.12.22
OQ821592	Cuba	16.12.22
OQ821593	Cuba	16.12.22
OQ821594	Cuba	18.12.22
OQ821595	Cuba	18.12.22
OQ821596	Cuba	18.12.22
OQ821597	Cuba	21.12.22
OQ821598	Cuba	22.12.22
OQ821599	Cuba	22.12.22
OQ821600	Cuba	01.01.23
OQ821601	Jamaica	11.06.16
OQ821602	Jamaica	27.07.16
OQ821603	Jamaica	12.09.16
OQ821604	Jamaica	15.01.19
OQ821605	Jamaica	01.02.19
OQ821606	Jamaica	15.07.19
OQ821607	Jamaica	12.08.19
OQ821608	Jamaica	19.10.19
OQ821609	Jamaica	06.11.19
OQ821610	Jamaica	17.01.20
OQ821611	Saint Lucia	07.11.20
OQ821613	USA	08.08.22
OQ821614	USA	11.08.22
OQ821615	USA	17.08.22
OQ821616	USA	04.10.22
OQ821617	USA	10.10.22
OQ821618	USA	15.10.22
OQ821619	USA	17.11.22
OQ821620	USA	21.11.22
OQ821621	USA	28.11.22
OQ821622	USA	11.12.22
OQ821623	USA	23.12.22
OQ747070	Cuba	18.07.22
OQ747071	Cuba	16.08.22
OQ721955	India	2022
OQ721956	India	2022
OQ721957	India	2022
OQ721958	India	2022
OQ721959	India	2022
OQ721960	India	2022
OQ721961	India	2022
OQ721962	India	2022
OQ721963	India	2022

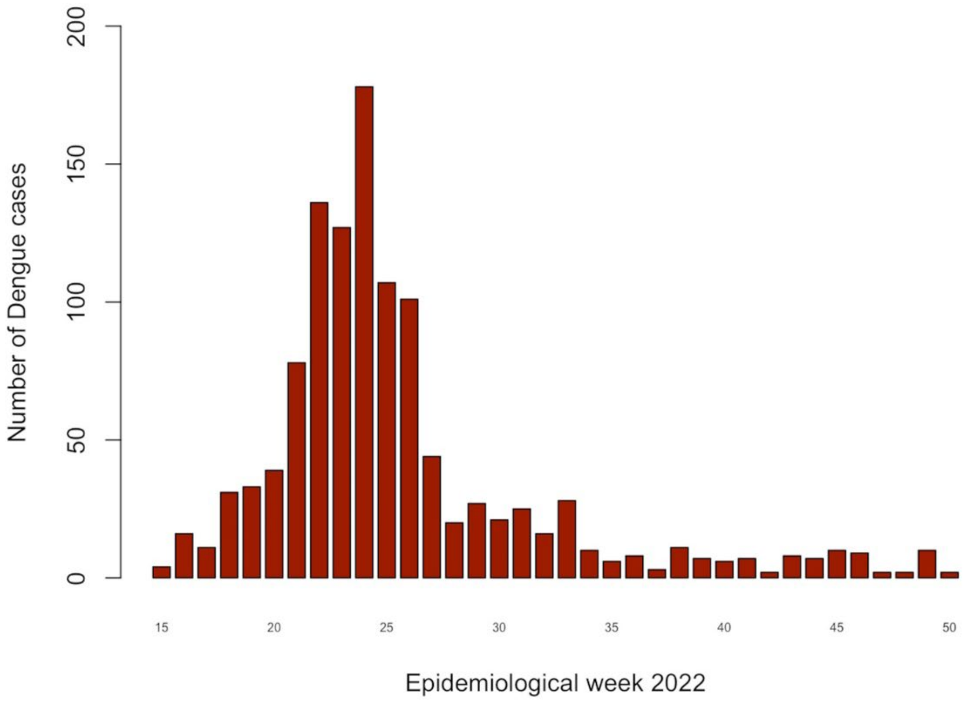

Appendix Figure 1. Epidemiological curve of the Dengue virus outbreak in the Democratic Republic of São Tomé and Príncipe, 2022. Notified Dengue disease cases were confirmed by a positive rapid test.

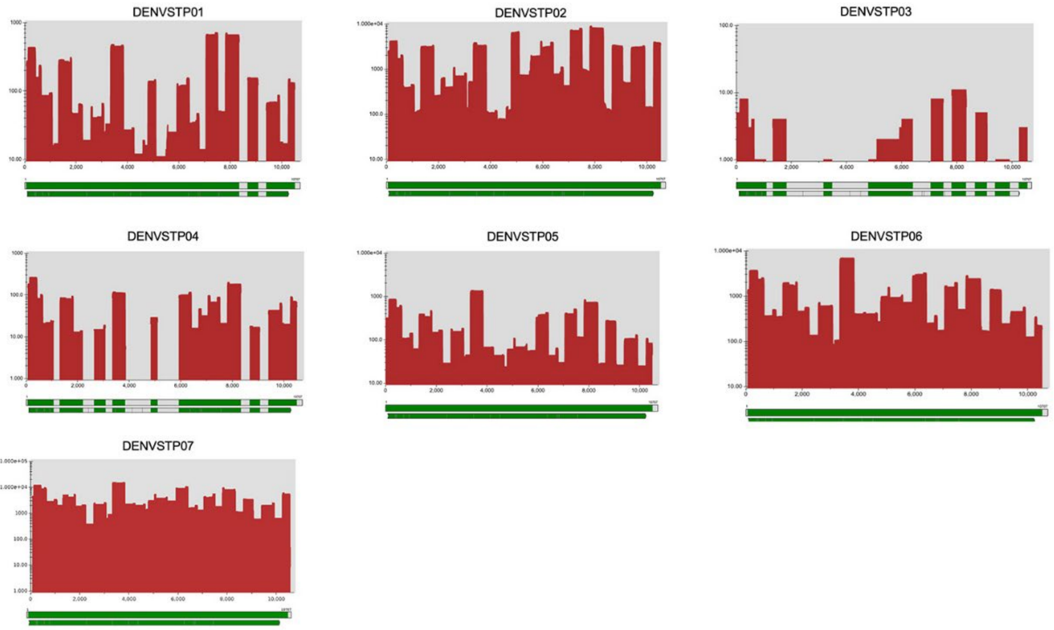

Appendix Figure 2. Genome coverage plots for seven sequenced DENV-3 GIII isolates from the Dengue virus outbreak in São Tomé and Príncipe, 2022. Assembled to reference NC_001475.

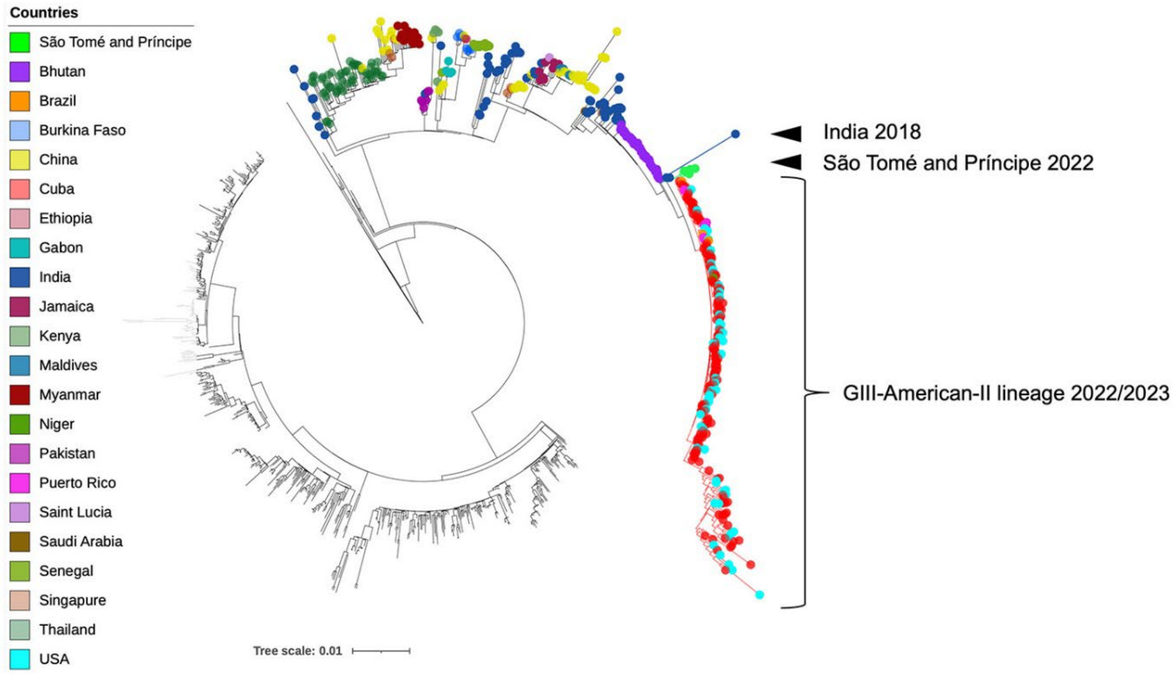

Appendix Figure 3. Unrooted phylogenomic tree including the four DENV-3 genomes from the Dengue outbreak in São Tomé and Príncipe in 2022 and 1,168 DENV-3 G III genomes sampled worldwide. The four STP sequences are clustered and branched between virus genomes isolated in India 2018 (dark blue) and the branch of the recent GIII-American-II lineage comprising isolates from Cuba (red), Brazil (orange), USA (light blue), and Puerto Rico (pink). Scale bar indicates nucleotide substitutions per site.
